# Over-prescribing of antibiotics and imaging in the management of uncomplicated URIs in emergency departments

**DOI:** 10.1186/1471-227X-13-7

**Published:** 2013-04-17

**Authors:** K Tom Xu, Daniel Roberts, Irvin Sulapas, Omar Martinez, Justin Berk, John Baldwin

**Affiliations:** 1 Department of Family & Community Medicine, School of Medicine, Texas Tech University Health Sciences Center, Lubbock, TX, USA; 2 Department of Surgery, School of Medicine, Texas Tech University Health Sciences Center, Lubbock, TX, USA; 3 Pharm D. Candidate, School of Pharmacy, Texas Tech University Health Sciences Center, Lubbock, TX, USA; 4 CHRISTUS Spohn Hospital - Memorial, Department of Emergency Medicine, Texas A&M Health Science Center, 2606 Hospital Blvd.,, Corpus Christi, TX 78405, USA

## Abstract

**Background:**

Unnecessary use of resources for common illnesses has substantial effect on patient care and costs. Evidence-based guidelines do not recommend antibiotics or imaging for uncomplicated upper respiratory infections (URIs). The objective of the current study was to examine medical care providers’ compliance with guidelines in treating uncomplicated URIs in emergency departments (EDs) in the US.

**Methods:**

Nationally representative data from the NHAMCS 2007 and 2008 were used. Uncomplicated URIs were identified through ICD-9 codes of nasopharyngitis, laryngitis, bronchitis, URI not otherwise specified and influenza involving upper respiratory tract. Exclusion criteria were concurrent comorbidities, follow-up visits, and age < 18 or >64 years. Most frequently prescribed classes of antibiotics were identified. Multivariate analyses were conducted to identify the factors associated with the prescribing of antibiotics and use of imaging studies.

**Results:**

In 2007 and 2008, there were 2.2 million adult uncomplicated URI visits without any other concurrent diagnoses in EDs in the US. Approximately 52% were given antibiotic prescriptions, over one-third of which were macrolides, and nearly half of the visits performed imaging studies. About 51% had a diagnosis of bronchitis, 35% URI NOS, 9% nasopharyngitis, laryngitis or influenza, and 4% multiple URI diagnoses. The diagnosis of bronchitis, fever at presentation, older ages, male gender, longer waiting time, and metropolitan areas were associated with a greater likelihood of prescribing antibiotics or imaging studies, controlling for confounding factors.

**Conclusion:**

Despite the recommendations and campaign efforts by the CDC and many medical associations, the prescribing of antibiotics in treating uncomplicated URIs in the EDs remains prevalent. Furthermore, overutilization of imaging studies is prevalent. Changes at levels of health care system and hospitals are needed to avoid unnecessary resource utilization. In addition, further patient education about antibiotic use in the community may greatly facilitate the transition out of an antibiotic-dependent consumer culture.

## Background

Inappropriate testing and treatments can lead to substantial over-expenditure in managing uncomplicated illnesses. Acute upper respiratory tract infection (URI) is one of the most common diagnoses seen in emergency departments (EDs) in the US. Between 1995 and 2000, there was an average of 8.5 million annual URI visits to the EDs [1], representing about 8% of all ED visits. In 2001–2002, about 23.7% of all ambulatory care visits were related to URIs [2]. To reduce the high prevalence of inappropriate treatment of uncomplicated URIs, the Centers for Disease Control and Prevention (CDC) and other medical organizations published guidelines for appropriate management.

The most studied aspect of the guidelines was the use of antibiotics. Several studies in the ambulatory care setting [3-5] and EDs [1] found that antibiotics were inappropriately used in over 50% of cases in the late 1990s and early 2000s. The study specific to EDs estimated the prevalence of inappropriate antibiotic use to be around 57% of adult URI visits [1]. One study showed that although the overall prevalence of antibiotic use was decreasing over time, the prescription of broad-spectrum antibiotics was on the rise [2]. The same study also concluded that the prevalence rates were comparable between EDs, office practices and outpatient clinics.

Imaging, especially plain radiography, of chest, has been utilized extensively in diagnosing respiratory diseases because of its availability, convenience and low cost. Several studies have demonstrated that unless a clinician suspected pneumonia or pathologies other than an uncomplicated URIs, imaging did not have additional diagnostic values after a thorough history and physical examination [6-9]. Consequently, CDC guidelines recommended that no routine diagnostic tests or imaging were needed without other indications in the outpatient management of uncomplicated URIs.

The objective of the current study was to examine medical care providers’ compliance with CDC guidelines in treating uncomplicated URIs in EDs in the US. The results provided benchmarks of providers’ compliance and insights into more effective and efficient management of uncomplicated URIs in emergency departments.

## Methods

Nationally representative emergency department data from the National Hospital Ambulatory Medical Care Survey (NHAMCS) 2007 and 2008 were used. Key data elements included patient demographic characteristics, visit characteristics, vital signs, tests, procedures, medications, discharge status and up to 3 diagnoses in ICD-9 codes. Details of the NHAMCS can be found at the CDC website (http://www.cdc.gov/nchs/ahcd.htm).

Uncomplicated URI diagnoses included ICD-9 codes for nasopharyngitis, laryngitis, bronchitis, URI NOS, and influenza involving upper respiratory tract. Several considerations were taken in selecting URI visits for the analyses. First, concurrent diagnoses of infections other than URIs, for example, urinary tract infection, could make the use of antibiotics appropriate. Second, antibiotic use could be appropriate for some upper respiratory infections, for example, sinusitis and otitis media. Third, concurrent chronic and acute diseases and conditions could justify the use of more aggressive treatments and diagnostic tests. Lastly, management strategies were different among pediatric, adult, and elderly patients.

Based on these considerations and the sample selection procedures used in previous studies of URIs using the NHAMCS data, the following inclusion and exclusion criteria were used to identify adult patients presenting with only uncomplicated URIs:

### Inclusion

Diagnoses of nasopharyngitis or common cold (ICD-9 460), laryngitis (ICD-9 464), bronchitis (ICD-9 466 and 490), URI NOS (ICD-9 465) and influenza that involves upper respiratory tract (ICD-9 487.1).

### Exclusion

Any concurrent comorbidities in the recorded diagnoses

Follow-up visit of a prior ED visit

Age < 18 or >64 years

The dependent variables were 1) the prescription of antibiotics and 2) the prescription of imaging studies (X-ray and CT). Several factors that may be associated with the prescription of antibiotics and imaging were investigated. The factors were selected *a priori* based on results from previous studies and the availability of information in the NHAMCS data. The types of URIs were categorized as URI NOS, nasopharyngitis, laryngitis, bronchitis, influenza, and multiple URI diagnoses. Nasopharyngitis, laryngitis and influenza were combined due to small cell sizes. Vital signs at presentation included whether the temperature was >100.4 Fahrenheit, whether the patient had tachydcardia (heart rate > 100 beats per minute) and whether the patient had systolic blood pressure (SBP) > 160 mmHg or diastolic blood pressure (DBP) >100 mmHg. Less than 20 patients had bradycardia (hear rate < 60 beats per minute) and pulse oximetry < 92%. Due to the concern of small cell sizes, bradycardia and oxygen saturation were not examined.

Characteristics of a visit included whether a patient presented with moderate or severe pain, waiting time to see a provider longer than 2 hours, whether a physician saw the patient, and the season. Patient demographic characteristics examined were age (41–64 vs. 18–40 years of age), sex, race, and ethnicity. Sources of payment were private insurance, Medicare, Medicaid, self-pay, and others. Sources of payment were not mutually exclusive because a patient may have multiple insurance types, for example, Medicare and private insurance. Geographic characteristics were Metropolitan Statistical Areas (MSA) and region (Northeast, Midwest, South and West).

To achieve a sufficient sample size, 2007 and 2008 were combined, as done in previous studies of URIs using NHAMCS. The complex sampling design was controlled for in all analyses to provide nationally representative estimates. Statistical software SAS® (SAS Institute, Cary, NC) and Stata ® (StataCorp LP, College Station, TX) were used to perform the analyses. First, the prescribing patterns of antibiotics and imaging were estimated. The most frequently prescribed antibiotic classes were then identified. Two multivariate logistic regressions were performed for prescribing antibiotics and imaging studies, respectively, to identify the effect of each independent variable, controlling for the confounding factors.

## Results

In 2007 and 2008, there were 241 million ED visits in the US, out of which 2.2 million were adult uncomplicated URIs without any other concurrent diagnoses. Among all uncomplicated URI visits, about 52% (95% CI: 47-58%) had antibiotic prescriptions and 46% (95% CI: 40-52%) had X-ray. Less than 2% of the visits performed CT studies. Approximately 51% (95% CI: 46-57%) of these visits had a diagnosis of bronchitis, 35% (95% CI: 29-40%) had a diagnosis of URI NOS, 9% (95% CI: 5-10%) had nasopharyngitis, laryngitis or influenza, and 4% (95% CI: 2-7%) had multiple URI diagnoses.

**Figure 1:**
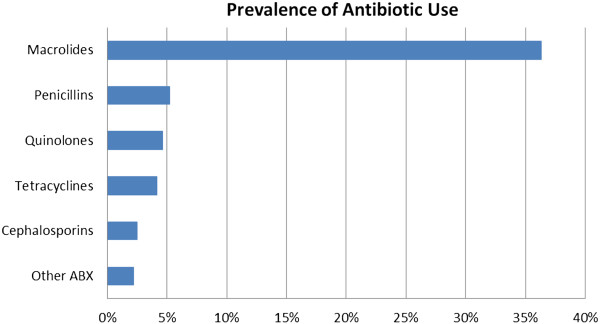
Prevalence of antibiotic use.

Figure 1 reports the prescription rate of each antibiotic class. About 36% (95% CI: 31-42%) of the visits included macrolide prescriptions, with the vast majority being azithromycin. Roughly 5% (95% CI: 3-8%) had penicillin prescriptions, almost all of which were amoxicillin and amoxicillin/clavulanate. Nearly 5% (95% CI: 3-7%) prescribed quinolones, the most frequent being levofloxacin followed by moxifloxacin and ciprofloxacin. Approximately 4% (95% CI: 3-6%) used tetracyclines, almost all of which were doxycycline.

Descriptive statistics of the sample characteristics are shown in Table 1. Table 2 reports results from the multivariate analyses. Among statistically significant (*p* < 0.05) findings, the diagnosis of bronchitis and multiple URI diagnoses were more likely than URI NOS to be associated with antibiotic prescriptions. In addition, the diagnosis of bronchitis was more likely than the diagnosis of URI NOS to be associated with the ordering of imaging studies. Among vital signs, fever was found to be significantly associated with a higher likelihood of prescribing antibiotics.

**Table 1:** Descriptive statistics (n = 616)

	**Unweighted sample**	**Weighted%**
**URI type**		
URI NOS	195	34.90
Nasopharyngitis, laryngitis, or influenza	57	9.27
Bronchitis	334	51.48
Multiple URI Dx	30	4.36
**Vital signs at presentation**		
Temperature > 100.4	58	8.90
Tachycardia	160	28.82
SBP > 160 or DBP > 100	54	8.00
**Characteristics of visit**		
Moderate or severe pain	292	49.49
Waiting time > 2 hrs	69	10.45
Not seen by a physician	72	13.32
Season		
Fall	123	18.04
Spring	168	27.59
Summer	84	16.39
Winter	241	37.98
**Demographics**		
41-64 years of age (vs. 18–40)	214	33.36
Female	387	65.78
Race/ethnicity		
Non-hispanic white	333	55.20
Non-hispanic black	186	29.86
Other races and ethnicities	97	14.95
**Source of payment**		
Private insurance	241	37.23
Medicare	46	8.00
Medicaid	158	24.77
Self pay	152	26.35
Other source of payment	56	9.33
**Geographic location**		
Metropolitan statistical areas	513	78.38
Region		
Northeast	164	20.88
Midwest	119	19.48
South	255	45.66
West	78	13.98

**Table 2:** **Results from multivariate analyses (odds ratios)**^***a ***^**(n = 616)**

	**Antibiotics**	**Imaging**
**URI type**		
URI NOS (Referent)		
Nasopharyngitis, laryngitis, or influenza	0.434	0.517
Bronchitis	11.333*	3.608*
Multiple URI Dx	4.796*	1.017
**Vital Signs at presentation**		
Temperature > 100.4	2.777*	1.450
Tachycardia	0.674	1.038
SBP > 160 or DBP > 100	0.670	1.044
**Characteristics of visit**		
Moderate or severe pain	1.212	1.436
Waiting time > 2 hrs	1.917*	1.116
Not seen by a physician	1.348	0.589
Season (Fall as referent)		
Spring	0.733	1.504
Summer	0.718	1.508
Winter	0.746	1.461
**Demographics**		
41-64 years of age (vs. 18–40)	1.432	2.300*
Female	0.464*	1.326
Race/ethnicity (Non-hispanic white as referent)		
Non-hispanic black	0.961	1.131
Other races and ethnicities	1.012	1.119
**Source of payment**		
Private insurance	0.491	3.532*
Medicare	0.356	2.982*
Medicaid	0.715	2.303
Self pay	0.558	1.618
Other source of payment	0.597	2.557
**Geographic location**		
Metropolitan statistical areas	0.835	2.295*
Region (Northeast as Referent)		
Midwest	1.101	1.044
South	1.814	0.934
West	0.773	0.754

*: *p* < 0.05.

^*a*^: Odds ratios are reported.

Waiting time longer than 2 hours was significantly associated with increased odds of prescribing antibiotics. As compared with males, female patients were found to be less likely to get antibiotics. Middle-aged patients were more likely than their younger counterparts to receive imaging studies. Having private insurance or Medicare was significantly associated with imaging studies. MSA status was significantly associated with increased probabilities of receiving imaging studies.

## Discussion

In spite of research evidence and guidelines on the management of uncomplicated URIs, over-prescribing of antibiotics and imaging studies, particularly plain radiography, persists. In 2000, over half of all ED visits for URIs had an antibiotic prescribed [2]. In 2005–2006, the antibiotic prescription rate reach 64% of URI visits in EDs [5]. Our study offered another piece of evidence that the overutilization of antibiotics in EDs continued into 2008, despite the growing concerns for antibiotic resistance and rising health care costs.

An early study reported that penicillins (13.1%), macrolides (25.8%), and cephalosporins (6.2%) were the most frequently prescribed antibiotics in 1995–2000 [1]. In 2005–2006, the prescription rate for penicillins and cephalosporins significantly decreased whereas that for macrolides increased from 1995–1996 [3]. Our study found that in 2007 and 2008, macrolide was the most frequently prescribed antibiotic class (35%) and penicillins were prescribed to only about 5% of the visits. Because of the differences in inclusion/exclusion criteria among the studies, the prescription prevalence rates are not directly comparable. However, it is evident that there have been changes in providers’ prescribing patterns.

The over-prescribing of macrolide, mostly azithromycin, may be attributable to its availability, low cost and microbial coverage. About 10% of adult pharyngitis cases are caused by Group A Hemolytic Streptococcus (GABHS). The most common organisms that cause community-acquired pneumonia include Streptococcus pneumoniae and Mycoplasma penumoniae. These three organisms, if not resistant, are sensitive to azithromycin. With the overlap of the symptoms of community-acquired pneumonia and upper respiratory infections, the increased use of azithromycin may be the result of diagnostic uncertainty and a shot-gun approach to treat the common respiratory symptoms seen in the EDs. Over time, many ED providers may have adopted this practice in treating the otherwise healthy patient population as a means to expedite patient disposition in response to increasing ED crowding and longer patient turn-over time.

The prescribing of antibiotics may also be associated with providers’ attempt to increase patient satisfaction [10-14]. In a study of 5 urban teaching hospital EDs, more treatments received in EDs was associated with a higher level of patient satisfaction, even after controlling for other confounding factors [15], although another study failed to demonstrate this association [16]. The current study found that longer waiting time was associated with prescribing antibiotics. This may have reflected ED providers’ efforts to prevent patient dissatisfaction rather than their propensity to prescribing antibiotics, for it was well demonstrated that waiting time in the EDs was a key predictor of patient satisfaction [15,17-20].

In addition, the current study found that almost half of the URI visits had imaging studies, particularly X-ray. Because we selected the healthiest age group without concurrent conditions from the general ED patient population to construct the sample, such a high prevalence of the use would suggest overutilization of care. One of the key rationales for ordering imaging studies among patients with respiratory symptoms was to rule out pneumonia. The incidence of community acquired pneumonia among patients with respiratory symptoms was between 2.7% and 7% in the general population [21-24]. For adult 18–64 years of age without comorbidities, the incidence rate should be much lower.

The overuse of imaging raises the concern for radiation-linked cancers, longer visits, and higher visit cost. Perhaps the overutilization of imaging mirrors the current trend of favoring diagnostic tests over more subjective clinical skills in all specialties of medicine. Over the past years, ED physicians have become accustomed to the core measures of pneumonia treatment implemented by the Joint Commission and the Centers for Medicare & Medicaid Services (http://www.jointcommission.org/pneumomia). Because of the heightened awareness of the burden of missed diagnosis of pneumonia, it is possible that ED physicians may have extended the use of imaging appropriate for a population at higher risks, i.e., the elderly population, to a younger and healthier age group.

There are several limitations in the current study. First, readers need to exercise cautions in comparing the prevalence rates obtained from the current study with those from prior studies due to different inclusion/exclusion criteria. The current study aimed to examine providers’ compliance with CDC guidelines. Consequently, the simplest form of URIs without any comorbidities in the healthiest population (18–64 years of age) were selected to eliminate justifiable deviations from the guidelines. As the result, the current study may have underestimated the prevalence of antibiotic and imaging prescriptions in the overall population and the results cannot be generalized to the pediatric and elderly populations. This also highlights the need of expanding the guidelines to encompass prevalent comorbidities, particularly those affecting respiratory, cardiovascular and immune systems. As the prevalence of chronic conditions grows in the US population, more research and concerted efforts are warranted to refine the existing URI treatment guidelines to curtail the over-prescribing of antibiotics and imaging studies in the EDs. Second, physical findings were not present in the data; ordering of some of the tests may have been appropriate if certain physical findings, for example, crackles heard upon auscultation, were taken into consideration. Third, any limitations or inconsistences in the ICD-9 coding of patient visits would lead to biases that are inherent in all studies involving coding.

Despite the recommendations and campaign efforts by the CDC and many medical associations, the prescribing of antibiotics in treating uncomplicated URIs in the EDs remains prevalent. Furthermore, overutilization of imaging studies is prevalent. Changes at levels of health care system and hospitals are needed to avoid unnecessary resource utilization. In addition, further patient education about antibiotic use in the community may greatly facilitate the transition out of an antibiotic-dependent consumer culture.

## Conclusion

Despite the recommendations and campaign efforts by the CDC and many medical associations, the prescribing of antibiotics in treating uncomplicated URIs in the EDs remains prevalent. Overutilization of imaging studies is also prevalent. Changes at levels of health care system and hospitals are needed to avoid unnecessary resource utilization. In addition, further patient education about antibiotic use in the community may greatly facilitate the transition out of an antibiotic-dependent consumer culture.

### Ethics

The data used for this study were obtained from a public domain, CDC’ website, http://www.cdc.gov/nchs/ahcd/ahcd_questionnaires.htm.

### Consent

The data used for this study were obtained from a public domain, CDC’ website, http://www.cdc.gov/nchs/ahcd/ahcd_questionnaires.htm.

## Competing interest

The authors declare that they have no competing interests.

## Authors’ contributions

KTX and JoB conceived the study. KTX performed data analyses. KTX, DR, IS, OM, JuB and JoB contributed to literature review, the interpretation of the results and the writing of the manuscript. All authors read and approved the final manuscript.

## Pre-publication history

The pre-publication history for this paper can be accessed here:

http://www.biomedcentral.com/1471-227X/13/7/prepub
